# Antimicrobial packaging based on ɛ‐polylysine bioactive film for the control of mycotoxigenic fungi in vitro and in bread

**DOI:** 10.1111/jfpp.13370

**Published:** 2017-06-09

**Authors:** C. Luz, J. Calpe, F. Saladino, Fernando B. Luciano, M. Fernandez‐Franzón, J. Mañes, G. Meca

**Affiliations:** ^1^ Laboratory of Food Chemistry and Toxicology, Faculty of Pharmacy University of Valencia, Av. Vicent Andrés Estellés s/n Burjassot, 46100 Spain; ^2^ School of Life Sciences Pontifícia Universidade Católica do Paraná Curitiba Paraná Brasil

## Abstract

ɛ‐Poly‐l‐lysine (ɛ‐PL) is a cationic peptide with a broad‐spectrum antimicrobial activity. This study investigates the use of ɛ‐PL as natural antimicrobial to inhibit fungal growth and to reduce aflatoxins (AFs) production. Antifungal activity of starch biofilms with different concentrations of ɛ‐Poly‐l‐lysine (ɛ‐PL) was determined in solid medium against *Aspergillus parasiticus* (AFs producer) and *Penicillium expansum*. Then, biofilms were tested as antimicrobial devices for the preservation of bread loaf inoculated with *A. parasiticus* CECT 2681 and *P. expansum* CECT 2278. Shelf life and AFs content were examined. Biofilms with concentrations of ɛ‐PL less than 1.6 mg/cm^2^ showed no fungal growth inhibition in solid medium, while the antifungal activity of the films with greater than 1.6 mg/cm^2^ of ɛ‐PL was dose dependent. The shelf life of bread inoculated with *A. parasiticus* was increased by 1 day with the use of films containing 1.6–6.5 mg ɛ‐PL/cm^2^, while shelf life of bread tainted with *P. expansum* was increased by 3 day with 6.5 mg ɛ‐PL/cm^2^. AFs production was greatly inhibited by ɛ‐PL biofilms (93–100%). Thus, ɛ‐PL biofilms could be potentially used as antimicrobial device during bread storage as a natural alternative to the synthetic preservatives.

**Practical applications:**

Ɛ‐Polylysin is a natural substance from microbial metabolism. Polylysine has a function to prevent a microbe from proliferating by ionic adsorption in the microbe. ɛ‐polylysine has a wide antibacterial spectrum and has an obvious lethal effect on Gram‐positive and Gram‐negative bacteria, yeast, mold, viruses, etc. It has a good antibacterial effect on the Gram‐negative bacteria *E. coli* and *Salmonellae*, which are difficult to control with other natural preservatives. ɛ‐Polylysine has already been used generally as a food additive in Japan, Korea and other part of world. In the United States, FDA has recognized the polylysine as a GRAS material. Considered the positive results obtained in the study, this compound could be used for the production of antimicrobial biofilms, applied as separator slices in the loaf bread production, to prevent the growth of the mycotoxigenic fungi *A. parasiticus* and *P. expansum*, contributing to reduce the use of the synthetically preservatives in bakery industry and also of the negative impact that these compounds could generate on the health of the end users.

## 1 INTRODUCTION

Preservatives are used to prolong food shelf life by eliminating/reducing the levels of pathogens or extending the time for spoilage microorganisms to reach unacceptable levels manifested as slime, visible colonies, compromised food texture, off‐flavors, and off‐odors resultant from microbial metabolism (Gram et al., 2002). Natural antimicrobials have presented high potential as food preservatives, reducing the growth bacteria and fungi alike. Moreover, the activity of these compounds can be further enhanced by controlling their release from food packaging or using encapsulation techniques (Hyldgaard, Mygind, & Meyer, 2012; Najjar, Kashtanov, & Chikindas, 2007; Sánchez‐González, Vargas, González‐Martínez, Chiralt, & Cháfer, 2011).

ɛ‐Poly‐l‐lysine (ɛ‐PL) is a cationic peptide produced by *Streptomyces albulus*, and it is composed by 25–35 l‐lysine residues linked through their carboxyl and ɛ‐amino groups rather than a conventional peptide bond. Shih, Shen, and Van (2006) studied the potential application of ɛ‐PL, among them its use as food preservative. ɛ‐PL has a broad‐spectrum antimicrobial activity against yeasts, molds, and Gram‐positive and Gram‐negative bacteria (Chang, Lu, Park, & Kang, 2010; Miya et al., 2014; Yoshida & Nagasawa, 2003). ɛ‐PL is positively charged at pH less than 9.0, which is common for most types of foods. Its cationic nature enables the interaction with negatively charged surfaces such as the cell surfaces of bacteria and fungi alike. This interaction results in membrane damage and consequent leakage of cytoplasmic content (Shima, Fukuhara, & Sakai, 1982; Shima, Matsuoka, Iwamoto, & Sakai, 1984). Hyldgaard et al. (2014) studied the ɛ‐PL's mechanism of action using *Escherichia coli* and *Listeria innocua* as model organisms. Although ɛ‐PL is not permitted as a food additive in most countries, the Food and Drug Administration of the United States has granted the status of generally recognized as safe to this product (FDA, 2011).

Some fungi that colonize agricultural commodities and foodstuffs may produce toxic secondary metabolites known as mycotoxins. Mycotoxin contamination in foods and feed is reported to be a global problem (Rodrigues & Chin, 2012; Rodrigues, Handl, & Binder, 2011; Rodrigues & Naehrer, 2012a, 2012b) and occur in approximately 25% of all grains produced worldwide (Wagacha & Muthomi, 2008). These fungal toxins produce serious health problems to humans and animals, including instant death in acute intoxication and cancer, immunosuppression, lack of growth and reproductive disorders with chronic exposure (Probst, Njapau, & Cotty, 2007; Varga, Frisvad, & Samson, 2009). In addition, significant economic losses result from mycotoxin contamination due to decreased market value of contaminated products and performance reduction in food animals fed with contaminated feed (Wu, 2006, 2007).

The objectives of this work were: (a) to study the antifungal activity in solid medium of ɛ‐PL biofilms against *Aspergillus parasiticus* and *Penicillium expansum*; (b) to determine the inhibition of fungal growth and to estimate the shelf life in breads preserved with ɛ‐PL biofilms and inoculated with *P. expansum* and *A. parasiticus*; (c) to evaluate the reduction in aflatoxin (AF) production (%) in bread inoculated with *A. parasiticus* and treated with ɛ‐PL biofilms.

## 2 MATERIALS AND METHODS

### 2.1 Materials

AFs B_1_, B_2_, G_1_, G_2_, formic acid (HCOOH), ammonium formate, sodium chloride (NaCl), corn starch, and glycerol were obtained from Sigma‐Aldrich (St. Louis, MO). Methanol was purchased from Fisher Scientific (NH). Deionized water (<18 MΩ cm resistivity) was obtained from a Milli‐Q water purification system (Millipore, Bedford, MA). Chromatographic solvents and water were degassed for 20 min using a Branson 5200 (Branson Ultrasonic Corp., CT) ultrasonic bath. Buffered peptone water, potato dextrose agar (PDA), phosphate buffer saline (PBS, pH 7.4), potato dextrose broth (PDB), De Man Rogosa Sharpe (MRS broth and agar) were provided by Oxoid (Madrid, Spain). The ɛ‐PL was provided by Bainafo (Henan, China). The strain of *A. parasiticus* CECT 2681 and *P. expansum* CECT 2278 were obtained from the Spanish Type Culture Collection (CECT, Valencia, Spain). These microorganisms were maintained in sterile glycerol at −80 °C. Then, they were recovered in PDB broth at 25 °C for 48 hr prior to use.

### 2.2 Methods

#### 2.2.1 ɛ‐Poly‐l‐lysine biofilm preparation

A 2% starch solution (w/v) was prepared by dissolving an appropriate amount of starch in water. The mixture was constantly stirred and heated from room temperature (∼25 °C) to 95 °C, and then held at 95 °C for 30 min. The solution was cooled down to room temperature, added with glycerol (0.25% w/v) as plasticizer and different concentrations of ɛ‐PL. Then, the mixture was homogenized by using an Ultra Ika T18 basic Ultraturrax (Staufen, Germany) at 5,000 rpm for 1 min. Aliquots of 10 mL of the prepared solutions were transferred to 55 mm Petri dishes, which were incubated for 48 hr at 37 °C. Finally, the dried films were carefully removed from the Petri dishes. ɛ‐PL final concentrations in the biofilms were expressed as mg of ɛ‐PL per cm^2^ of biofilm: 0, 0.2, 0.4, 0.8, 1.6, 3.2, and 6.5 mg ɛ‐PL/cm^2^.

#### 2.2.2 Antifungal activity of biofilms

The disc‐diffusion method was used to evaluate the antimicrobial activity of ɛ‐PL biofilms (Madhyastha, Marquardt, Masi, Borsa, & Frohlich, 1994). *A. parasiticus* and *P. expansum* were cultured on 9 mm Petri dishes containing PDA and incubated for 7 days at 30 °C. Then, 1 mL of sterile saline solution was added on the agar surface and spores were collected with a pipette. The suspension was adjusted to 10^6^ spores/mL. Aliquots of 0.1 mL were used to contaminate new PDA plates through spread plating. Biofilms with different concentrations of ɛ‐PL were placed on the agar surface just after inoculation. Petri dishes were refrigerated at 4 °C for 6 hr to allow the bioactive compounds to diffuse into the agar, and then incubated 7 days at 30 °C. According to Castlebury, Sutherland, Tanner, Henderson, and Cerniglia (1999), the microorganisms were considered positive to the antimicrobial activity of the bioactive compounds if an inhibition zone of at least 8 mm in diameter was observed around the disc.

#### 2.2.3 Baking and bread treatment with polylysine biofilms

Bread recipe included 600 g of wheat flour, 20 g of sucrose, 10 g of NaCl, 40 g of bakery yeast (Levital, Spain), and 350 mL of distilled water. Ingredients were mixed manually for 5 min and the dough produced was left rising for 1 hr at room temperature. Baking was performed at 230 °C for 30 min in an electric oven (MIWE, Arnstein, Germany). The oven was pre‐steamed (300 mL of water) before cooking. Loaves were kept for 30 min on cooling racks at room temperature before they were cut in circular slices of 10 g each. The slices were inoculated in four equidistant points with 10 µl of a suspension containing 1 × 10^5^ spores/mL of *P. expansum* CECT 2278 or *A. parasiticus* CECT 2681. Spores were counted using a Neubauer chamber and adjusted to 10^5^ spore/mL in buffered peptone water as reported by Kelly, Grimm, Bendig, Hempel, and Krull (2006). Then, ɛ‐PL biofilms of four different concentrations (0, 1.6, 3.2, 6.5 mg polylysine/cm^2^) were placed on the inoculated circular slices. The slices were introduced in 1 L plastic bags that were thermally sealed (Sammic TS‐150, Basarte, España) and stored at room temperature for 7 days. Bread slices were examined daily and their shelf life was considered as the first day when visible fungal growth was noticed. At day 7, all packages were opened and samples were used to determine the AFs content by liquid chromatography coupled to mass spectrometry in tandem (LC‐MS/MS).

#### 2.2.4 Aflatoxins extraction

AF extraction was performed using the method described by Hontanaya, Meca, Luciano, Mañes, and Font (2015). Briefly, the bread slices were finely ground with a blender (Oster Classic grinder, Oster, Valencia, Spain) and 5 g samples were placed in 50 mL plastic tubes. Then, 25 mL of a methanol were added and samples were homogenized using Ultra Ika T18 basic Ultraturrax (Staufen, Germany) for 3 min. The mixture was centrifuged at 4,500 rpm for 5 min and the supernatant was evaporated to dryness with a Büchi Rotavapor R‐200 (Postfach, Switzerland). The residue was re‐dissolved in 1 mL of extraction solvent, filtered through a 0.22 µm syringe filter and injected to the LC‐MS/MS system.

#### 2.2.5 AFs identification and quantification by LC‐MS/MS

LC‐MS/MS analyses were performed with an Agilent 1200 chromatograph (Agilent Technologies, Palo Alto, CA) coupled to a 3200 QTRAP mass spectrometer (Applied Bio‐systems, AB Sciex, Foster City, CA) equipped with a turbo ionspray electrospray ionization (ESI) interface. The instrument data were collected and processed using the Analyst version 1.5.2 software. Separation of analytes was performed using a reversed‐phase analytical column (Gemini C18 column, 150 × 2 mm, I.D.; 3 µm particle size) equipped with a security guard cartridge C18 (4 × 2 mm, I.D.; 5 µm) purchased from Phenomenex (Madrid, Spain). The mobile phase was composed of solvent A as water + 0.1% formic acid and solvent B as methanol + 0.1% formic acid. Both solvents used as mobile phase also contained 5 mM of ammonium formate. The gradient elution was established initially with 10% B, increased to 90% B in 4 min and kept constant during 6 min. The eluent B was increased to 100% in 1 min, kept constant 1 min and then decreased to 50% in 2 min. Afterward, the initial conditions were reestablished in 2 min and maintained for 4 min.

The flow rate was 0.25 mL/min and MS/MS was achieved in the selected reaction monitoring (SRM) mode using ESI in positive mode. For LC‐MS/MS analysis, scheduled SRM was used with a 120 s SRM detection window and 1 s of target scan time. The applied parameters were: ion spray voltage, 5500 V; source temperature, 450C; curtain gas, 20; ion source gas 1 (sheath gas), 50 psi; ion source gas 2 (drying gas), 55 psi. Nitrogen served as nebulizer and collision gas. The ionization and fragmentation parameters used for the detection and quantification of the AFs were set according to Liu et al. (2013).

#### 2.2.6 Determination of the fungal population

Bread samples (10 g) were transferred to a sterile plastic bags containing 90 mL of sterile peptone water (Oxoid, Madrid, Spain) and homogenized with a stomacher (IUL, Barcelona, Spain) during 30 s. The mixture was serially diluted in sterile plastic tubes containing 0.1% peptone water. Aliquots of 0.1 mL were plated in acidified (pH 3.5) potato dextrose agar (Insulab, Valencia, Spain) and the plates were incubated at 25 °C for up to 7 days prior to microbial counting (Pitt & Hocking, 1997).

## 3 RESULTS AND DISCUSSION

### 3.1 Antifungal activity of polylysine films

**Figure 1:**
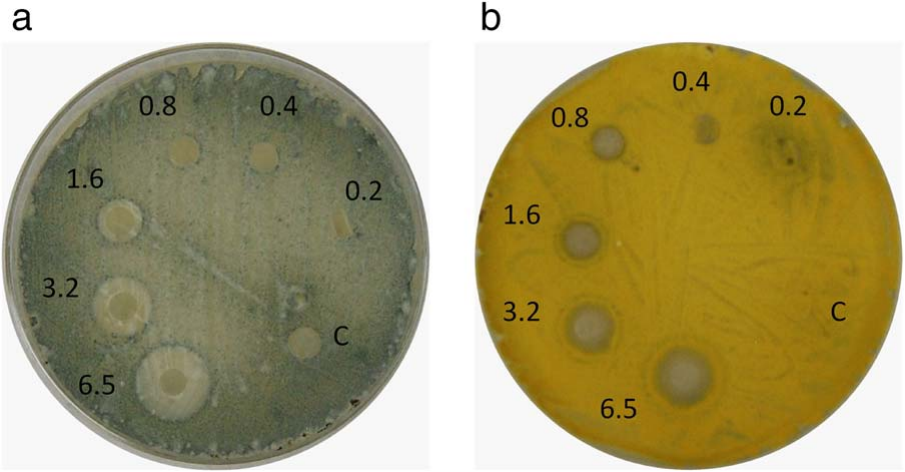
Antifungal activity of ɛ‐PL biofilms against (a) *P. expansum* and (b) *A. parasiticus*

The results obtained for antifungal activity of ɛ‐PL biofilms at different concentrations (0.2–6.5 mg/cm^2^) against *A. parasiticus* and *P. expansum* inoculated in Petri dishes are shown in Figure 1. Inhibitory activity against *A. parasiticus* was observed in biofilms with concentration of ɛ‐PL between 0.8–6.5 mg/cm^2^ whereas concentrations ranging from 1.6 to 6.5 mg/cm^2^ resulted efficient against *P. expansum*. The antifungal activity of the ɛ‐PL biofilms was dose dependent and biofilms with less than 0.8–1.6 mg/cm^2^ showed no fungal growth inhibition.

Hata, Sato, Ichikawa, and Morimitsu (2016) have observed the minimum inhibitory concentration of ɛ‐PL added directly to PDA plates. *Penicillium* sp. WA1‐1 and *Aspergillus niger* WA2‐4 growth were inhibited with 0.2% (w/v) of ɛ‐PL. Starch film containing ɛ‐PL has been prepared before and its activity has been tested against *Escherichia coli*, *Bacillus subtilis*, and *Aspergillus niger* (Zhang et al., 2015). Unfortunately, it is practically impossible to calculate the concentration of ɛ‐PL per cm^2^ in these films and compare with the results found in the present study. The authors have added ɛ‐PL to the starch solution after gelatinization and concentrations were described as % of ɛ‐PL in 100 g of starch. Nonetheless, 2% ɛ‐PL films were able to form inhibition zones for all organisms tested.

### 3.2 Shelf life improvement and aflatoxins reduction in bread

**Figure 2:**
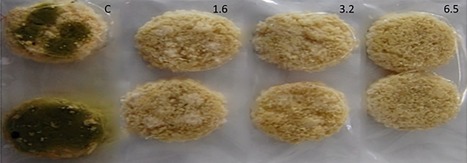
Growth inhibition of (a) *P. expansum* and (b) *A. parasiticus* in breads preserved with ɛ‐PL biofilms at different concentrations (polylysine mg/cm^2^). C = control

**Figure 3:**
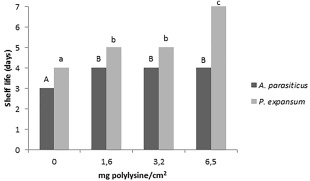
Shelf life of bread preserved with biofilms of ε‐PL at different concentrations and inoculated with *P. expansum* and *A. parasiticus*

Results of the shelf life study of breads loaves preserved with ɛ‐PL films are shown in Figures 2 and 3. All films could significantly increase the shelf life loaves inoculated with either *A. parasiticus* or *P. expansum* (Figure 3). ɛ‐PL biofilm containing 6.5 mg polylysine/cm^2^ was able to increase the shelf life of bread samples inoculated with *P. expansum* in three days, while biofilms with 1.6 and 3.2 mg polylysine/cm^2^ delayed fungal growth by one day in comparison with control samples. On the other hand, the shelf life of bread inoculated with *A. parasiticus* was increased by 1 day if compared with the control, independently of the ɛ‐PL concentration used (Figure 3).

**Figure 4:**
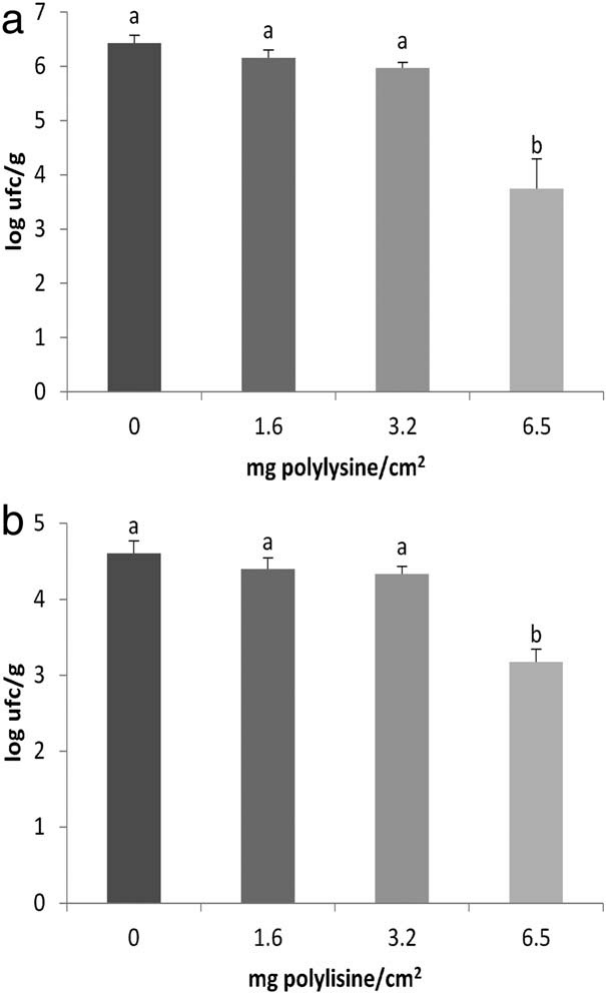
Reduction of mold growth in bread inoculated with (a) *P. expansum* and (b) *A. parasiticus* treated with ε‐PL biofilms at different concentrations, after 7 days of storage. Different letters show significant difference (*p* ≤ .05) among treatments

Although the shelf life of bread was improved by all treatments with biofilms containing ɛ‐PL, only films with 6.5 mg polylysine/cm^2^ were able to significantly reduce the population of *P. expansum* (2.73 log CFU/mL) and *A. parasiticus* (1.42 log CFU/g) in comparison with the control after 7 days of storage (Figure 4).

Considering that the strain of the *A. parasiticus* used in this study was AFs producer, the control and treated bread were extracted for AFs detection and quantification using LC‐MS/MS. The method was validated for all AFs studying linearity, recovery, repeatability, reproducibility, LODs and matrix effect. The coefficients of determination (*R* ^2^) were always higher than .9923. The matrix effects obtained ranged between 27 and 37%. The recoveries evidenced ranged between 84 and 88%. Intra‐day (*n* = 3) and interday (3 different days) variation values ranged between 2.6 and 4.2%. The detection limit (LOD) and the limit of quantification (LOQ) values were calculated according to *s/n* = 3 and *s/n* = 10, respectively. The LODs were 0.08 μg/kg for AFB_1_ and AFB_2_, 0.16 μg/kg for AFG_1_ and 0.30 μg/kg for AFG_2_. The LOQs were 0.27 μg/kg for AFB_1_ and AFB_2_, 0.53 μg/kg for AFG_1_ and 1 μg/kg for AFG_2_.

Results of AFs analysis by LC‐MS/MS confirmed that ɛ‐PL biofilms reduced the production of AFs in comparison with the control. Control samples presented 2607.57 µg/kg of AFB_1_, 7.00 µg/kg AFB_2_, 622.91 µg/kg AFG_1_, and 17.17 µg/kg AFG_2_. Table 1 shows the AFs reduction (%) observed in bread treated with ɛ‐PL biofilms after 7 d. With 1.6 mg polylysine/cm^2^ the reduction was above 95% for AFB_1_ and above 97% for AFB_2_, AFG_1_ and AFG_2_; using 3.2 mg polylysine/cm^2^ the reduction was greater than 93% for AFB_1_ and greater than 97% for the other AFs; with 6.5 mg polylysine/cm^2^ the reduction was greater than 97% for all AFs. There was only a significant difference among doses for AFB_1_ reduction, where 6.5 mg polylysine/cm^2^ (97.59%) showed higher activity than the other concentrations. Similar reduction profile was obtained with all concentrations for other AFs.

**Table 1:** AFs reduction (%) in breads preserved with ε‐PL biofilm and inoculated with *A. parasiticus*

	% Reduction AFs			
mg polylysine/cm^2^	AFB_1_	AFB_2_	AFG_1_	AFG_2_
**1.6**	95.31 ± 0.54	97.71 ± 0.06	97.80 ± 0.03	98.92 ± 0.14
**3.2**	93.90 ± 2.80	97.00 ± 1.38	97.46 ± 1.59	99.28 ± 1.14
**6.5**	97.59 ± 0.96	98.88 ± 0.35	99.30 ± 0.43	100.00

To our knowledge, this was the first time that a biodegradable film containing ɛ‐PL was used to combat the growth of mycotoxigenic molds in bread. Other authors have also incorporated natural antimicrobials to different films targeting fungal growth inhibition in bread. Cinnamon and clove powder (0.06–0.40 g/100g) were incorporated in biodegradable films based on cassava starch to preserve slices of white bread (Kechichian, Ditchfield, Veiga‐Santos, & Tadini, 2010). After incubation for 7 days at 25 °C, the authors did not report any inhibition of the growth of yeasts and mold. In contrast, nano‐emulsions of 40 mg/mL clove bud and oregano essential oils (EOS) incorporated in methylcellulose films reduced yeast and mold counts on sliced bread stored at 25 ± 2 °C (Otoni, Pontes, Medeiros, & Soares, 2014). The authors reported a decrease in the counts after 15 days of storage with the films as compared with the control, and demonstrated a significant enhancement of the growth inhibition by using nano‐emulsions of clove bud EO compared with coarse emulsions. In contrast, no significant effect of the type of emulsion was exhibited for oregano EO. A cellulose‐derivative polymer dissolved in acetone and 5 or 10% cinnamaldehyde was used to obtain films for the preservation of bread (Lopes et al., 2014). After 12 days of storage at 23 ± 2 °C, yeast and mold counts were lower than 2 log CFU/g, compared with greater than 6 log CFU/g found in the control group. It has been suggested that the antimicrobial activity of EOs can be the result of the enzymatic cell system damage, including enzymes associated with the production of energy and with the synthesis of structural components (Tian et al., 2011). It has also been reported that the effect of phenolic compounds on microbial growth and the production of toxins could be due to the ability of these compounds to alter microbial cell permeability. ɛ‐PL also acts in the cellular membrane changing its permeability and, since mycotoxins are secondary metabolites, *A. parasiticus* may direct its metabolism for other defense mechanisms in the presence of the natural antimicrobial.

## 4 CONCLUSIONS

ɛ‐PL biofilms inhibited *A. Parasiticus* and *P. expansum* growth on solid medium. In bread they showed antifungal activity against both fungi but biofilms were more efficient against *P. expansum*. Shelf life of bread inoculated with *P. expansum* could be increased in 3 days using biofilm with 6.5 mg of ɛ‐PL/cm^2^, while 1.6 and 3.2 mg of ɛ‐PL/cm^2^ increased the shelf life by 1 day. All concentrations were able to increase the shelf life of bread contaminated with *A. parasiticus* by 1 day, but AFs reduction was greater than 93.90% for all concentrations of ɛ‐PL tested after 7 days, greatly improving the safety of this product. These properties of ɛ‐PL biofilms potentially allow them to be used as antimicrobial devices during storage of bread, and maybe other food, as a natural alternative to the synthetic preservatives.
